# Single-sex schistosomiasis: a mini review

**DOI:** 10.3389/fimmu.2023.1158805

**Published:** 2023-04-19

**Authors:** Haoran Zhong, Yamei Jin

**Affiliations:** ^1^ National Reference Laboratory for Animal Schistosomiasis, Shanghai Veterinary Research Institute, Chinese Academy of Agricultural Sciences, Shanghai, China; ^2^ Key Laboratory of Animal Parasitology of Ministry of Agriculture and Rural Affairs, Shanghai Veterinary Research Institute, Chinese Academy of Agricultural Sciences, Shanghai, China

**Keywords:** schistosome, single-sex infection, omics, host-parasite interaction, immune regulation

## Abstract

Schistosomiasis is a neglected tropical disease caused by dioecious blood flukes of the genus *Schistosoma* and second to malaria as a parasitic disease with significant socio-economic impacts. Mating is essential for maturation of male and female schistosomes and for females to lay of eggs, which are responsible for the pathogenesis and propagation of the life cycle beyond the mammalian host. Single-sex schistosomes, which do not produce viable eggs without mating, have been overlooked given the symptomatic paucity of the single-sex schistosomiasis and limited diagnostic toolkit. Besides, single-sex schistosomes are less sensitive to praziquantel. Therefore, these issues should be considered to achieve the elimination of this infection disease. The aim of this review is to summarize current progress in research of single-sex schistosomes and host-parasite interactions.

## Introduction

Schistosomiasis is a neglected tropical disease caused by parasitic flatworms (blood flukes) of the genus *Schistosoma* that affects about 250 million people mainly in tropical and subtropical regions and accounts for 1.4–3.3 million disability-adjusted life years annually (1, 2). The World Health Organization (WHO) currently recommends mass administration of praziquantel (PZQ) for the control, worm/egg burden reduction and elimination of schistosomiasis (3). The increasing attention to schistosomiasis over the past few decades has led to significant improvements in agricultural and irrigation practices, as well as sanitation and hygiene (4, 5). Data from the national surveillance sites for schistosomiasis in China show that there have been no cases of *Schistosoma japonicum* infection of humans, bovines, or aquatic snails as the intermediate host in 2021 (6), demonstrating remarkable achievements in the control of schistosomiasis. The considerable expansion of preventive chemotherapy in tandem with scientific progress have improved the global control of schistosomiasis (2, 7). A publication by the WHO, titled “Ending the neglect to attain the Sustainable Development Goals: a road map for neglected tropical diseases 2021–2030”, proposes more ambitious targets, including the elimination of schistosomiasis as a public health threat (8). Nevertheless, as a highly complex multi-host parasite, many questions remain unexplored.

Dioecious schistosomes mate and lay eggs within either the mesenteric or venules of the plexus venous of urogenital organs (depending on the species) (1). Most eggs are excreted in urine or feces, thereby contributing to propagate the life cycle. The presence of eggs in faecal or urine samples is considered the gold standard diagnostic tool (9). However, this approach is insufficient for detection of single-sex schistosomiasis (10). An opinion article published in 2018 proposed that exposure to single-sex schistosomes is an overlooked phenomenon, as no eggs are produced within the final host and infection is asymptomatic (11). Most importantly, a single-sex infection cannot be identified by traditional egg-based parasitological tests, which undoubtedly jeopardizes diagnostic accuracy of schistosomiasis (11). Other methods to theoretically detect egg-free single-sex schistosome infection include monitoring of schistosome gut-associated circulating anodic and cathodic antigens in serum or urine (12), and real-time polymerase chain reaction (PCR) analysis of schistosome DNA (13). When considering cost and convenience of rapid detection, circulating cathodic antigen is a direction worthy of further study in the future (14).

Both field and laboratory studies have reported cases of single-sex schistosome infections (15–18). Theoretically, single-sex schistosomes only mate in the host after encountering a worm of the opposite sex (17, 19). However, schistosomes produced by single-sex infections can also mate with the opposite sex of heterologous species and produce viable eggs (20–25). Another recent study indicated that both male and female *S*. *japonicum* can survive treatment with PZQ and retain normal reproductive potential (26). Therefore, it is necessary to address infections of single-sex schistosomes. Elucidation of the mechanisms underlying reproductive development of schistosomes and host-parasite interactions offers new perspectives for drug administration and therapies.

## Single-sex schistosome infection occurs under natural conditions

Schistosomes mature only after mating and can subsequently survive in human hosts for 3–10 years (27, 28). Initial studies suggested that single-sex schistosome infections do not occur in nature but they can be generated in laboratory animals with cercariae produced from snails infected with single-sex miracidia (29, 30). However, subsequent field studies have found that single-sex schistosome infections do indeed occur in nature (15).

Single-sex miracidia can infect the tropical freshwater snail *Oncomelania hupensis* as the intermediate host (31). A field study conducted in the hilly areas of Anhui, China identified 67 (0.78%) of 8563 snails infected with schistosomes (32). Of the 46 snails selected for further studies, 21 (45.7%) were infected with female schistosomes, 23 (50.0%) with males, and only 2 (4.3%) with both sexes (32). Other field studies have reported similar results for *S*. *mansoni* and *S*. *haematobium* (31, 33). Besides, an ecological survey of definitive hosts conducted in 1993 found that 43 (20.8%) of 207 wild rats were infected with single-sex schistosomes, which included 38 (88.4%) infected with males and 5 (11.6%) with females (18). Another survey conducted along the Yangtze River (Hubei, China) identified 22 (5.5%) of 400 sentinel mice infected with schistosomes, of which 14 (63.6%) were infected with only males and 2 (9.1%) with only females (15). However, there is a lack of data from field investigations on how long single-sex schistosomes can survive in the host. Besides, schistosomes have numerous naturally permissive and non-adaptive hosts, thus surveillance is extremely difficult, which can potentially limit control measures (1). Moreover, single-sex schistosomes can remain “mating-ready” for up to 1 year in mice and still produce viable eggs (17, 34). These findings provide compelling proof that schistosomes can exist unpaired in natural environments.

## Morphological differences between single-sex and bisexual worms

Morphological observations after dissection are commonly used to identify infections with single-sex schistosomes (35). Single-sex female (SF) worms and bisexual mated female (MF) worms have distinct morphological differences. In general, SF worms are about one-third of the length of MF worms, which renders detection relatively difficult (36). In *S. japonicum*, developed ovaries could be observed in MF worms at 18 day post infection (dpi) (2–3 days after mating) (37). From 21–25 dpi, MF worms continue to develop with proliferation of vitelline cells, while the ovaries and vitelline glands of SF worms were stunted which contains only stage I vitellocytes (38). An *in vitro* study found that when separated from the male, the female will lose the ability to produce viable eggs due to the loss of mature oocytes (39). Unlike females, there is no significant morphological difference between single-sex infected male worms (SM) and bisexual infected mated male worms (MM) (40, 41), although the testes of SM worms are slightly smaller (16).

## Current omics studies of single-sex schistosomes

Laboratory models for stable single-sex infections can be established by infecting snails with single miracidia and identifying the sex of the cercariae released by individual snails by PCR (42). This model of single-sex infection has paved the way towards a better understanding of single-sex schistosomiasis in the mammalian host (42, 43).

Omics studies are useful to explore the reproductive development of schistosomes. Recent developments of sequence databases and improvements in proteomics and transcriptomics technologies have facilitated high-throughput studies of single-sex schistosomes and provided useful references for further studies of the reproductive mechanisms (44, 45).

### Transcriptomics

Alexis et al. (29) identified differentially expressed genes (DEGs) between 42-day *S*. *mansoni* SF and MF worms. The results of *in situ* hybridization studies found that DEGs were mainly localized in the vitellocytes and ovary of MF worms vs. the vitellocytes and subtegumental cells of SF worms (29). The various predicted functions of DEGs in MF worms include oocyte maturation, apoptosis, protein degradation, and interactions between vitellocytes (29). The predicted functions of the DEGs 6767 (GenBank no. CCD61090) and 15402 (GenBank no. XP_002573676) of SF worms involve interactions that occur during mating (46).

An in-depth study conducted by Lu et al. (47) applied RNA-sequencing (RNA-seq) analyses for comparisons of isolated complete ovaries and testes from paired (46-day) and unpaired (67-day) *S*. *mansoni* and the adult worm. The results identified 96 genes comparatively enriched in testes of MM worms and 147 in the testes of SM worms. However, the mating procedure resulted in nearly 15-fold more differentially expressed genes (DEGs) in the ovaries between SF and MF worms (47). This enormous discrepancy in quantity is probably, as mentioned above, closely related to the intuitive morphological differences (16). Reference to the Kyoto Encyclopedia of Genes and Genomes classified the DEGs to metabolic and regulatory pathways (48). The results revealed that 849 DEGs from the ovaries of MF worms were involved in the Akt-, MAPK-, and Ras-signaling pathways, ribosome biogenesis, RNA transport, and endocytosis, whereas 435 DEGs from the ovaries of SF worms were involved in focal adhesion, lysosome function, and the MAPK signaling pathway (47). The schistosome egg-shell precursor gene p14 (Smp_131110), which was up-regulated in MF worms, encodes a female-specific tyrosinase that plays a pivotal role in egg shell synthesis (49). Notably, the transcript profiles of SF worms were more similar to either SM or MM worms than MF worms, which might be associated with the evolutionary background (50).

A meta-analysis of RNA-seq studies offered valuable expression data across all life stages of *S*. *mansoni* (51). In addition, an interactive web portal was established (51) of not only RNA-seq data, but also conserved structural domains and related pathways. Combined with the WormBase ParaSite database (52), this web portal allows researchers to visualize and analyze data for genomic studies of schistosomes.

Single-cell RNA-seq has also been used to comprehensively describe tissue types and physiology of schistosomes (53). A study by Wendt et al. (53) classified 43642 cells from the adult *S. mansoni* into 68 distinct cell populations. These data will help to further clarify the development of various cellular lineages during the schistosome life cycle to facilitate the development of novel therapeutics.

### Proteomics

Proteomics has been used extensively in schistosomiasis research and has facilitated the discovery of critical molecules involved in reproductive development and as potential vaccine targets (16, 37, 45, 54–58). Schistosomes are highly complex organisms, thus dynamic analysis of the developmental stages is warranted (59). Based on the life cycle of *S*. *japonicum* (mating at 15 or 16 dpi, eggshell formation at 22 dpi, and egg laying at 24 dpi) (1), the proteomic profiles of SF and MF worms at 18, 21, 23, and 25 dpi were elucidated by our group (37, 45). In total, 2835 differentially expressed proteins (DEPs) were identified between SF and MF worms at different developmental stages (37). Relative to SF worms, 402, 322, 415, and 505 DEPs were up-regulated, while 230, 267, 290, and 404 DEPs were down-regulated in MF worms at 18, 21, 23, and 25 dpi, respectively (37). Gene ontology functional annotations demonstrated that 34 DEPs down-regulated in MF worms at all four time points were mainly involved in actin-related cell cycle-related functions, whereas 44 DEPs up-regulated in MF worms were involved in protein folding and hydrolysis, redox reactions, translation, and calcium ion binding (37, 60, 61). *Schistosoma japonicum* translationally controlled tumor protein (SjTCTP), which was highly expressed in MF worms at 18, 21, 23, and 25 dpi, is essential to the development of *S*. *japonicum* and recombinant SjTCTP was reported to stimulate partial protective immunity against schistosome infection in BALB/c mice (37).

Similarly, our group conducted comparative analysis of the proteomic profiles of SM and MM worms at 18, 21, 23, and 25 dpi (16), which confirmed 674 DEPs at different developmental stages. As compared to studies of female worms, there were significantly fewer DEPs both overall and at single time points, similar to the transcriptome changes described above (47). However, the proteomics and transcriptomics profiles were not always consistent, possibly because of differences in the timing and loci of gene transcription and translation or posttranscriptional regulation of proteins, suggesting that these differences were not coincidental (62, 63). Interestingly, bioinformatics analysis identified some DEPs closely associated with tumor proliferation in mammals, suggesting that the biological function of a protein might have similar functions in different tissues or organisms and could possibly be involved in the growth and differentiation of some cells in schistosomes (64, 65). *S. haematobium* is classified as a class I carcinogen by the International Agency for Research on Cancer that is associated with squamous cell carcinoma of the urinary bladder. Proteomic studies that reveal the functional role of DEPs during tumour development in the context of urogenital schistosomiasis are critical to identify not only targets for control, but biomarkers. Promising proteomic studies looking at *S. haematobium* worm tegument and soluble egg proteins validate this statement (66).

### miRNomics: MicroRNA biology and computational analysis

MicroRNAs (miRNAs) are small non-coding RNAs that can negatively regulate the expression of target genes at the post-transcriptional level by binding to the 3’- and 5’-untranslated regions and coding sequences in order to repress translation or initiate degradation (67). Schistosome-derived miRNAs have been implicated in schistosome development and host-parasite interactions in schistosomiasis (68–70). The miRNA expression profiles of SF and MF *S*. *japonicum* have been reported (71, 72). Sun et al. (71) investigated differentially expressed miRNAs of 18- and 23-day SF and MF, and found similar miRNA profiles in 18-day SF and MF worms, whereas in 23-day MF worms, sja-bantam was significantly up-regulated, while sja-miR-1, sja-miR-7, sja-miR-7-5p, and sja-miR-71 were significantly up-regulated in 23-day SF worms (71). The predicted target genes of sja-bantam are reportedly related to development of the embryo and primary sexual characteristics, while the four up-regulated miRNAs in 23-day SF worms are associated with ribonucleoprotein complex assembly and microtubule-based processes (71, 73). A previous study by our group identified differentially expressed miRNAs between 25-day SF and MF worms (72), where sja-bantam and 19 other miRNAs were up-regulated in 25-day MF worms, while sja-miR-1, sja-miR-7-5p, and 14 other miRNAs were up-regulated in 25-day SF worms (72). Furthermore, comparisons of the expression profiles of sma-miR-277, sma-miR-4989, and related target genes in single-sex infected schistosomes suggested that sma-miR-277 and sma-miR-4989 have pivotal roles during juvenile-to-adult transition (74). Collectively, these findings indicate that differentially expressed miRNAs and related target genes might regulate the sexual status of female worms.

Although omics has provided tremendous insights into the mystery of single-sex schistosomes, relatively few studies have investigated epigenetics and small non-coding RNAs, other than miRNAs. A summary of recent studies of single-sex infected schistosomes is provided in **Table 1**.

**Table 1:** Studies involving schistosome single-sex infections in the last 30 years.

Year	Natural/ Artificial	Species	Main content	Reference
1993	Natural	*S. mansoni*	Distribution of SF and SM within the host population	(18)
1994	Artificial	*S. japonicum*	Circulating antigen detection of SF and SM infection	(19)
1994	Artificial	*S. mansoni &* *S. intercalatum*	Single-sex schistosomes mated with the opposite sexes of heterologous species	(21)
1995	Artificial	*S. mansoni &* *S. haematobium*	Single-sex schistosomes mated with the opposite sexes of heterologous species	(20)
1995	Artificial	*S. mansoni &* *S. intercalatum*	Mating competition between males	(22)
1999	Artificial	*S. mansoni &* *S. haematobium*	Single-sex schistosomes mated with the opposite sexes of heterologous species	(25)
2001	Artificial	*S. mansoni*	PCR for sexing cercariae	(43)
2002	Natural	*S. mansoni*	Single-sex infected snails found in the field	(33)
2002	Artificial	*Schistosoma mansoni &* *S. margrebowiei*	Single-sex schistosomes mated with the opposite sexes of heterologous species	(23)
2003	Artificial	*S. mansoni &* *S. intercalatum*	Single-sex schistosomes mated with the opposite sexes of heterologous species	(24)
2004	Artificial	*S. mansoni*	Morphological observation of SF and MF	(38)
2006	Artificial	*S. mansoni*	Transcriptional analysis of 49-day SF and SM	(73)
2012	Artificial	*S. mansoni*	Transcriptional analysis of 49-day SF and MF	(29)
2012	Natural	*S. japonicum*	Single-sex infected schistosomes found in surveillance	(15)
2014	Natural	*S. japonicum*	Single-sex infected snails found in the field	(32)
2014	Artificial	*S. japonicum*	MicroRNA profiles of 18- and 23-day SF and MF	(71)
2016	Artificial	*S. mansoni*	Transcriptional analysis of 67-day SF and SM	(47)
2017	Artificial	*S. mansoni*	Profiles of sma-mir-277/4989 and its target genes	(74)
2017	Artificial	*S. mansoni*	SF mitigates liver fibrosis after secondary infection	(75)
2018	Artificial	*S. mansoni*	Transcriptional analysis of all life stages of SF and SM	(51)
2018	/	/	Opinion article regarding the importance of single-sex infection	(11)
2018	Artificial	*S. mansoni*	Immunoregulation of SF and SM	(76)
2019	Artificial	*S. mansoni*	SF mitigates liver fibrosis after secondary infection	(77)
2020	Artificial	*S. japonicum*	Duplex RT-PCR for sexing cercariae	(42)
2020	Artificial	*S. japonicum*	Proteomic profiles of 25-day SF and MF	(45)
2020	Artificial	*S. japonicum*	MicroRNA profiles of 25-day SF and MF	(72)
2020	Artificial	*S. mansoni*	Single-cell RNA-seq analysis of 6–7 weeks SF and MF	(53)
2021	Artificial	*S. japonicum*	Survival and reproductive potential of SF and SM	(17)
2022	Artificial	*S. mansoni*	CCA and CAA of SF and SM	(12)
2022	Artificial	*S. japonicum*	Proteomic profiles of 18-, 21-, 23-, 25-day SF and MF	(37)
2022	Artificial	*S. japonicum*	Proteomic profiles of 18-, 21-, 23-, 25-day SM and MM	(16)
2022	Artificial	*S. japonicum*	PZQ treatment of SF and SM	(26)
2022	Artificial	*S. mansoni*	Transcriptional analysis of host infected with SF and SM	(78)
2022	Artificial	*S. mansoni*	BATT from male worms stimulate SF to develop	(79)
2023	Artificial	*S. japonicum*	Functional analysis of DEPs coming from SF and MF	(61)
2023	Artificial	*S. mansoni*	Single-sex infection boost immune response	(80)

SF, single-sex female worms; SM, single-sex male worms; MF, bisexual mated female worms; MM, bisexual mated male worms; PCR, polymerase chain reaction; RT-PCR, real-time polymerase chain reaction; CCA, circulating cathodic antigen; CAA, circulating anodic antigen; PZQ, praziquantel; BATT, β-alanyl-tryptamine; DEP, differentially expressed protein. /, not applicable.

## Single-sex schistosomes regulate hepatic fibrosis in the host

Prolonged survival of schistosomes in the host can facilitate regulation of the host immune response through intricate mechanisms (81). Briefly, a T helper type 1 (Th1) response was generated during the initial phase of infection, which could target immature and mature migrating parasites (82). Schistosomes eggs trigger a dominate Th2 response regulated by regulatory T-cells (Tregs) (81). Granulomatous hypersensitivity to eggs trapped in the liver and intestinal tissues trigger the fundamental pathological causation of schistosomiasis, which could also be interpreted as a strong repair response (83) to suppress inflammation during the initial infection, but can also lead to tissue fibrosis (84). Mature female *S*. *japonicum* and *S*. *mansoni* produce hundreds of eggs per day, of which some become trapped in the liver, leading to hepatic inflammation, granuloma formation and fibrosis, and portal hypertension that leads to ascites (85). Without prompt treatment, schistosome-induced hepatic fibrosis is often irreversible (86). Therefore, targeting regulation of the host immune response could be useful for treatment or even reversal of schistosome-induced hepatic fibrosis.

Recent studies have investigated the immunomodulatory mechanisms of single-sex schistosomes in schistosome-induced hepatic fibrosis (75, 76, 78). Nicole et al. (75). demonstrated that primary infection of female *S*. *mansoni* (week 0–11) in secondary bisexually infected mice (week 12–19, then sacrificed) resulted in suppression of Th2-mediated granuloma and hepatic fibrosis, although no change in parasite load was observed. Mice with a primary infection of male *S*. *mansoni* also showed signs of reduced fibrosis, though not as evident as those initially infected with females (75), possibly due to high expression of cytotoxic T-lymphocyte-associated protein 4 (Ctla4) by Foxp3+ Tregs (87). Besides, the lack of relatively high production of Th1 cells implies that Th1 and Th2 responses might be regulated independently (75). Subsequent experiments indicated that Ctla4 had a preventive effect against schistosome-induced hepatic fibrosis (77).

When the timing of two infections were varied (primary infection at week 0–6 and secondary infection at week 7–14), protection against unisexual infection was not achieved when reinfection occurred more than 6 weeks later, thereby revealing the roles of male schistosomes in immune regulation (76). Male schistosomes triggered strong Th2 innate immune reactions during recurrent infection, which led to the strong recruitment of innate inflammatory cells, especially neutrophils and eosinophils, eventually resulting in reduced burdens of worms and eggs (76). However, the reduction to the parasite load in mice with primary infection of male schistosomes had a limited effect on granuloma size and hepatic fibrosis, which might be related to the high level of Th2 cytokines, such as interleukin-13, which promotes fibrosis (88).

A recent comparative transcriptomic analysis has helped to clarify the differences in immune regulation between male and female schistosomes (78). In this study, the number of DEGs in the spleens of mice infected with male schistosomes was more than two-fold greater than mice infected with females, suggesting greater involvement of males (78). Further analysis found that male schistosomes drove dendritic cell maturation and induced T cell differentiation *via* up-regulation of the costimulatory molecule CD86, whereas infection with unisexual female worms had little effect on the host immune system (78).

**Figure 1:**
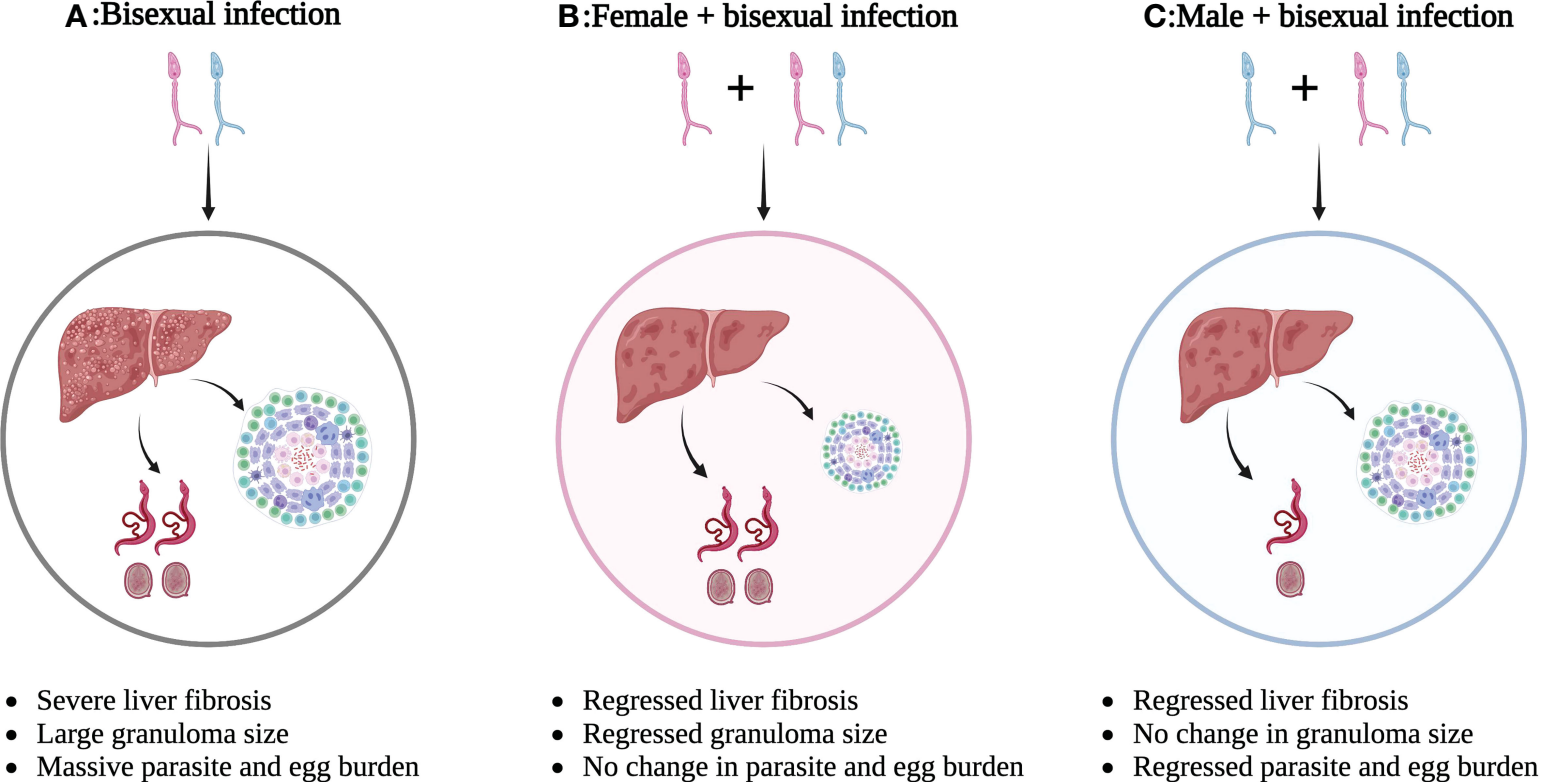
Different outcomes of single-sex schistosome-induced hepatic fibrosis. **(A)** Bisexual schistosome infection leads to severe liver fibrosis with large granuloma size and massive parasite/egg burden; **(B)** Bisexual schistosome infection after female infection leads to regressed liver fibrosis with smaller granuloma size, but no change in parasite/egg burden; **(C)** Bisexual schistosome infection after male infection leads to regressed liver fibrosis with regressed parasite/egg burden, but no change in granuloma size. This figure was created with Biorender.com. Arrows indicate the result of the next step.

Overall, infection by single-sex schistosomes (either sex) can induce immune responses in the host, which subsequently causes a Th1/Th2 imbalance, although further studies are needed to better clarify the effects of female schistosomes. These findings provide promising targets for new immune modulatory strategies against schistosome-induced hepatic fibrosis and possibly other diseases. The different outcomes of single-sex schistosome-induced hepatic fibrosis are presented in **Figure 1**.

## Challenges and future perspectives

Several unresolved challenges and problems remain due to insufficient knowledge of single-sex schistosomes, including (i) the application of traditional egg-based parasitological tests for detection of single-sex infections; (ii) detection of antigens or nucleic acids as diagnostic criteria, as positive results might be due to past schistosome exposure or cross-reactions, rather than a single-sex schistosome infection (89, 90), and the reliance on morphological observations after dissection, which can create logistical difficulties; (iii) greater resistance of long-lived single-sex schistosomes to PZQ than paired worms (26); and (iv) the application of Omics studies to identify potential diagnostic and therapeutic targets. Extracellular vesicles (EVs) released from schistosome eggs and worms at different developmental stages were identified and considered to be important vectors in the regulation of host-parasite interactions (70). Studies have reported that the cargo of schistosome-derived EVs include sja-miR-1 (91), sma-miR-10 (92), sja-miR-71a (69), sja-miR-125b (93), sja-miR-2162 (94), sja-bantam (93) and novel sja-miRNA-33 (95), which mediate cross-species host-parasite interactions. However, the expression profiles of the EVs of single-sex schistosomes remain unknown. Thus, further analysis is warranted to provide new insights into immune regulation by single-sex schistosomes.

The development of *in vitro* culture methods for schistosomes has helped to validate the reliability of targets screened from Omics studies (96). In fact, a recent transcriptomic study indicated that male schistosomes can stimulate synthesis of the pheromone β-alanyl-tryptamine *via* nonribosomal peptide synthetase to facilitate normal development and laying of eggs by female schistosomes cultured *in vitro* (79). The results of this study suggest avenues for therapeutic intervention and demonstrated the unlimited potential of single-sex schistosome research.

The recently proposed “hygiene hypothesis” has made it possible to apply the immunomodulatory effects of schistosomes for the prevention and treatment of autoimmune disorders, other than liver fibrosis (69, 94, 95, 97). Schistosome infection and schistosome-derived proteins, peptides, miRNAs and EVs have been reported for the treatment of human immune-related disorders, including allergic asthma (98), arthritis (99), colitis (100), diabetes (101), sepsis (102), cystitis (103), and cancers (104, 105). In addition, human models of male schistosome cercariae infection, which produced no eggs or associated pathology, may provide foundations for subsequent studies (12, 106, 107). Nonetheless, further studies are needed to elucidate the distinct immunomodulatory mechanisms of male and female schistosomes and potential impacts on human diseases.

## Conclusions

Current control measures to reduce the infection rate of snails and definitive hosts could greatly increase the incidence of infection with single-sex schistosomes. These measures target the egg load in definitive hosts to reduce infection of snails, but also increase the risk for snails becoming hosts to mono-miracidia and production of only one sex of cercariae that can infect the definitive host (80).

Exposure to single-sex schistosomes is a neglected phenomenon that may become a threat of the control and elimination of schistosomiasis as a public health issue. The continued development of diagnostic and treatment protocols is imperative and further investigations of the reproductive development of schistosomes, as well as single-sex schistosomes, may provide essential references. In addition, future studies of the interactions between single-sex schistosomes and the host are warranted as a theoretical basis for autoimmune disorders, other than schistosomiasis.

## Author contributions

YJ and HZ: conceptualization, validation, investigation, resources, and writing – review and editing. HZ: writing – original draft preparation and visualization. YJ: supervision, project administration, and funding acquisition. Both authors contributed to the article and approved the submitted version.
